# Randomised controlled trial of tourniquet associated pain generated in lower limb after exsanguination by Esmarch bandage versus limb elevation

**DOI:** 10.1186/s13018-024-04749-1

**Published:** 2024-05-03

**Authors:** Alexander Mitrichev, John Maunder, Aiden Jabur, Prince Singh, Deborah Lees, Levi Morse, Benjamin Parkinson

**Affiliations:** 1 Princess Alexandra Hospital Orthopaedic Department, 199 Ipswich Road, Woolloongabba, QLD 4102 Australia; 2 Faculty of Medicine, University of Queensland, Brisbane, Australia; 3 Townsville General Hospital Orthopaedic Department, Townsville, Australia; 4 Cairns Base Hospital Orthopaedic Department, Cairns, Australia; 5 Faculty of Medicine, James Cook University, Townsville, Australia

**Keywords:** Tourniquet, Pain, Lower limb

## Abstract

**Background:**

Tourniquets are common adjuncts in the operating theatre but can be associated with post-operative pain. This study was designed to compare what effect pre-tourniquet Esmarch bandage exsanguination has on pain, compared to pre-tourniquet exsanguination by elevation alone.

**Methods:**

52 volunteers (104 lower limbs) were included in this study with each volunteer acting as their own matched control. The primary outcome was patient reported pain, measured in both legs simultaneously using area under curve. Secondary outcomes were pain score during inflation and deflation, cumulative pain score, duration of recovery and blood pressure during testing.

**Results:**

Pain after Esmarch was superior to elevation as measured by area under pain curve (68.9 SD 26.1 vs 77.2 SD 27.3, *p* = 0.0010), independent of leg dominance. Cumulative pain scores demonstrated the same superiority after inflation (50.7 SD 17.1 vs 52.9 SD 17.0, *p* = 0.026) but not after deflation (*p* = 0.59). Blood pressure was not significantly different. Time to full recovery of the lower limb was the same for both groups—7.6 min (SD 2.1 min, *p* = 0.80).

**Conclusion:**

Previous studies describe a positive effect on pain when Esmarch bandage was used prior to tourniquet inflation for upper limb. Our findings suggest the same benefit from Esmarch when it was used on lower limbs—particularly during inflation of tourniquet. In addition to pain profiles, surgeon preference and patient factors need to be considered when deciding between elevation and Esmarch bandage.

## Introduction

Predating ancient Rome [1], the tourniquet has been widely used in upper and lower limb surgeries. Over time, it evolved from a simple hand-tied constricting band to the modern-day pneumatic tourniquet [2]. It restricts blood flow in the operative limb to allow better visualisation of surgical field and prevent excessive blood loss during surgery. However, prolonged tourniquet use is associated with pain, morbidity and mortality [3–7].

Compression from the tourniquet results in ischaemic cascade [8]. Decreased blood flow leads to inadequate oxygen delivery and impaired removal of metabolic waste products, including carbon dioxide and lactate, at the level of the tourniquet and distally [9]. As the result of this process, acidosis develops and progresses to tissue damage with time [10].

Ischaemic pain was found to be less intense if adequate exsanguination of the limb is performed prior to tourniquet inflation [11]. Surgeons are divided on the choice of limb exsanguination by elevation versus wrapped compressive bandage prior to tourniquet inflation. A clinical trial investigating the difference in pain level between these two methods for the upper limb found that exsanguination was more comfortable for the patient compared to simple elevation above the head, both during the tourniquet inflation period and afterwards in the recovery phase [12].

As of yet, no trials exist comparing methods of exsanguination prior to tourniquet inflation in the lower limb. We aimed to determine whether Esmarch bandage exsanguination or limb elevation before tourniquet application resulted in more discomfort both during inflation and after deflation. Our primary outcome was degree of patient reported pain measured by area under curve and secondary outcomes were pain at every 2-min check, cumulative pain score, duration of recovery phase and blood pressure during occlusion and release time.

## Patients and methods

This study was registered in the Australia and New Zealand Clinical Trials Registry (ACTRN12622000727741p) and ethically approved by the Townsville Hospital & Health Service (HREC/2022/QTHS/80081) to be carried out at Townsville University Hospital and Cairns Base Hospital.

This prospective single-blind randomised control trial involved each participant receiving the intervention (Esmarch bandage exsanguination) and control (limb elevation) measures simultaneously in opposite lower limbs.

### Recruitment

The study was conducted in the orthopaedic clinic at Townsville University Hospital and Cairns Base Hospital. Email invitations were sent to Queensland Health staff in both hospitals. One week was allowed for recruitment of study participants via email with information leaflet and consent form attached. A paper copy of the signed consent form was obtained on their day of testing. We aimed to match or exceed the number of participants used in a similar study conducted by Lees et al. on the upper limb-26 volunteers [12]. The eligibility of participants was determined using the inclusion and exclusion criteria in Table 1. Participants had the opportunity to withdraw from the study at any time.

**Table 1:** Inclusion and exclusion criteria

Inclusion criteria
Age over 18 years Ability to provide informed consent Attendance for study measurements
Exclusion criteria
Peripheral or central nervous system disorders, including acute or chronic brain pathology, compressive peripheral neuropathy, spinal canal stenosis or nerve root impingement Abnormal power or sensation in lower limbs Medical condition affecting blood supply or sensation of lower limbs such as diabetes, multiple sclerosis, peripheral vascular disease, myocardial infarction, stokes, hypertension, anaemia and preventative medications for whose conditions Any type of lower limb tendinitis Hip, knee or ankle dislocation in past 12 months Previous trauma to hip, knee ankle or foot resulted in deficit muscles power or sensation Thoracic, lumbar or sacral spine osteoarthritis Previous injury, trauma or surgery on thoracic, lumbar or sacral spine History of venous thromboembolism or arterial occlusion Surgical procedure performed on lower limbs in the last 12 months Medications Pharmacological medication, supplements or traditional medicines affecting coagulation profile

### Study methods

A minimum of four participants were scheduled per business day. Data collection occurred over 3 months. No formal long term follow up was required. Patients’ full name, age, sex and dominant limb was recorded. Initial pain score based on visual analogue scale (VAS) was recorded, as well as blood pressure and inspection of the skin. VAS pain score ranged from 0 to 10, with 0 being no pain and 10 being the greatest pain that the patient could imagine. Patients were pseudo-randomised by dominant/non-dominant limb using computer algorithm and informed of their randomisation via envelope in the clinic room. Once comfortable lying supine on the hospital bed, Softban Orthopaedic Wool (Smith & Nephew, London, United Kingdom) was applied to the limb undergoing Esmarch bandage exsanguination followed by the bandage itself. The other lower limb was simultaneously elevated to 45° of hip flexion for three minutes using a stirrup. Tourniquets were then applied to both thighs, simultaneously inflated to a standard pressure of 300 mmHg and the timer started. The SmartPump Dual Channel machine was used as tourniquet (Stryker Pty Ltd, Sydney, Australia, ARTG entry 266269). Researchers then immediately changed room for blinding purposes. VAS score was recorded at baseline prior to randomisation and every two minutes until the tourniquet was deflated and VAS score returned to baseline. The tourniquet was deflated either at 20 min or when the participant requested this due to abnormal discomfort, whichever came first (Table 2).

**Table 2:** Mean (SD) cumulative pain scores during inflation and deflation, area under curve and time to full recovery for elevated and Esmarch exsanguinated limbs

	Elevation	Esmarch	*p*-value
Area under curve	77.2 (27.3)	68.9 (26.1)	0.0010
Cumulative scores under inflation	52.9 (17.0)	50.7 (17.1)	0.026
Cumulative scores under deflation	24.1 (15.3)	23.4 (14.8)	0.59
Time until full recovery	7.6 (2.1)	7.6 (2.1)	0.80

### Outcome measures

The primary outcome was area under the pain curve after inflation and deflation. Secondary outcome measures were pain at every 2 min check, cumulative pain score after 20 min, duration of recovery phase and blood pressure (systolic blood pressure—SBP, diastolic blood pressure—SBP, and mean arterial pressure—MAP) throughout testing. Main potential adverse effects from tourniquet were pain, temporary loss of sensation and/or changes in skin colour below tourniquet, loss of muscle strength during inflation of tourniquet and early after deflation, elevation of blood pressure secondary to pain and bruise at the site of compression.

### Sample size

The determination of sample size required for this study used a power calculation identical to that used in the study by Lees, Penny and Baker [12]. Data from previous studies suggests that the expected mean area under pain curve would be approximately 80 (SD 25) using the assumption that the pain curve exhibits an initial sharp increase in pain, followed by a more gradual linear increase [12–14]. Using an effect size of 20% of the mean, a power of 0.8 and a p-value of 0.05, the minimum required sample size was 22 patients, or 22 lower limbs in each group.

### Statistical analyses

The data is reported as mean ± standard deviation. Q–Q plots and Kolmogorov–Smirnov tests were used to assess normality of outcome distribution and ensure the data met the assumptions of the statistical tests used. A 2-way (limb x time) repeated measures analysis of variance (ANOVA) was conducted for the pain measures. A one-way repeated measures analysis of variance was conducted for SBP, DBP and MAP. When an interaction effect, main effect of limb or time was identified, a Bonferroni pairwise comparison was conducted as post-hoc tests. For the area under curve and cumulative pain levels across the time points, a paired *t*-test was used to compare measures between each limb. Statistical analysis was performed using SAS 9.4 software (SAS Institute Inc., Cary, NC, USA). The alpha level was set as 0.05 for all tests.

## Results

104 lower limbs (52 participants) were used for this study, comprised of 50 female and 54 male lower limbs. Mean patient age was 28.4 years (SD 7.5 years). Seven patients stated that their dominant lower limb was the left—the remainder being right-footed.

**Fig. 1:**
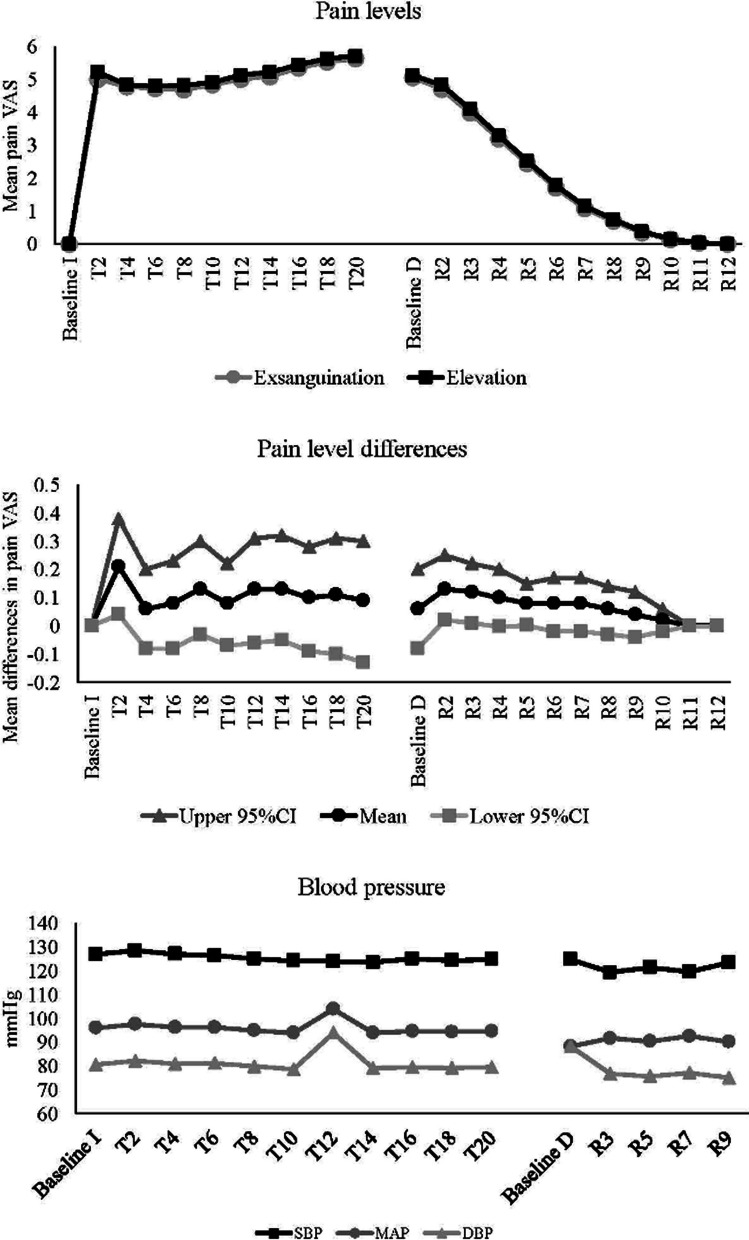
The pain levels for each limb (top), pain level differences between limbs (middle) and blood pressure measures (bottom) during tourniquet occlusion (T) and release (R)

Figure 1 depicts the pain levels for each limb, pain differences between limbs and blood pressure measures during occlusion (T, tourniquet inflated) and release (R, tourniquet released). ‘Baseline I’ is defined as the time point just prior to tourniquet inflation and’Baseline D’ is the time point just after tourniquet deflation. None of the participants opted to cease prior to 20 min. There was statistically significant improvement in pain with Esmarch limb exsanguination compared to exsanguination by elevation (area under curve 68.9 vs 77.2, *p* = 0.0010). There was also a main time effect, with pain increasing from Baseline I–T2, and then increasing again from T6 to T20. Similar trends were found for release, with neither interaction effect in regard to limb dominance nor main effect of limb exsanguination method on pain. However, the pain levels were reversed, with reductions at each time point, until the differences reached a plateau by R10.

There was significant improvement in cumulative pain score with Esmarch during tourniquet inflation, however not after release (Fig. 1). For blood pressure, there were no main time effects for DBP and MAP during occlusion. However, SBP was greater at T2 when compared to T12, T14 and T16 during inflation. DBP and hence MAP exhibited an unanticipated rise at T12 with no identifiable cause and may represent an anomaly in the data. During release, there was no main time effect for SBP and DBP. However, MAP was significantly greater at R3 when compared to R1. Time to full recovery was unchanged between the two groups.

## Discussion

In similar fashion to Lees et al. (2016), our findings suggest that Esmarch bandage exsanguination prior to tourniquet inflation for 20 min on the lower limb significantly improves pain compared to elevation exsanguination [12]. Cumulative pain scores after deflation were not significantly different between the two groups. Time until return to baseline (pre-tourniquet inflation) pain level in the limb was also unaffected by exsanguination method. A similar study conducted on the upper limb has shown that there was a statistically significant improvement in pain with Esmarch exsanguination (*p* = 0.004) when using the area under curve method of cumulative pain measurement [12].

Scintigraphy has shown that Esmarch bandage reduces regional blood volume in the lower limb by 64% and hence produces a greater exsanguination than limb elevation at 60° for 30 s which exhibited a 45% reduction [15]. Furthermore, the same study found that an extended period of limb elevation up to 10 min did not significantly change the blood volume reduction (44%) [15]. One previous study suggests that when blood is exsanguinated from the arm, ischaemic pain does not develop until 30–45 min have elapsed [16]. To our knowledge, no studies have explicitly associated the scintigraphic evidence of Esmarch exsanguination with decreased overall tourniquet pain. We theorise that the effect of intravascular and interstitial pressures may contribute to pain sensation in the limb, and that a more effective exsanguination method—as measured by scintigraphy—may therefore lead to less pain in comparison to less effective methods. The aetiology of tourniquet-related pain continues to be studied, however the effect of limb intravascular pressure on pain is not a new idea; studies on gravitational load applied in the head-to-foot direction have shown that limb pain results from the markedly increased intravascular pressure [17].

It is well described that angle of limb elevation is an important factor in exsanguination; stasis of the blood in the limb does not occur at 60° [13] but has been shown to occur at 90° due to obstruction of venous outflow [18].

Technique variables play an important role in how tourniquet pain is perceived by the patient. Duration of tourniquet inflation and use of regional anaesthesia has been found to be in direct proportion to pain [19]. However, inflation pressure did not significantly impact the incidence or severity of pain in the same study, regardless of whether the tourniquet was applied to the upper limb or lower limb [19].

Pain benefits aside, there are risks associated with Esmarch bandage use for exsanguination. Skin tension blisters are a documented adverse effect that occurs more commonly with this technique compared to limb elevation [20]. This is due to constant friction generated between the Esmarch bandage and the skin resulting in the accumulation of fluid-filled vesicles underneath the epidermis. They may become complicated with superficial or deep infections [21]. Other events such as wound haematoma, wound ooze, deep vein thrombosis (DVT) and pulmonary embolism (PE) are less common but have similar incidence rates between Esmarch and elevation [20]. Use of Esmarch bandage and tourniquet with subsequent mechanical dislodgement of pre-existing DVT causing PE has been recorded in the literature, with some cases even resulting in death [22–26]. The majority of these are from orthopaedic trauma cases, however some were recorded for elective cases such as total knee arthroplasty (TKR) [23]. In light of this potentially fatal outcome, some authors recommend against the use of Esmarch bandage for exsanguination in TKR [27, 28], knee arthroscopy [28], and trauma cases, particularly where there has been a delay for surgical treatment [22].

Blood pressure measurements did not contribute to the comparison between the two exsanguination methods due to the simultaneous testing, but instead gave a physiological measure of pain levels. Pain and the increase in systemic vascular resistance due to arterial occlusion are known to be factors raising blood pressure.

One strength of this study is that 52 participants volunteered—the largest study comparing tourniquet-associated pain between Esmarch and limb elevation to our knowledge. The paired design of this study—with patients reporting on pain levels for the two techniques simultaneously on each lower limb—lends greater intra-rater reliability. The recruitment of healthy volunteers with no upper limb pathology and a near-equal representation of male and female sexes reduced the influence of these factors known to affect pain perception. Furthermore, no surgical procedure was being undertaken at the time which may otherwise induce anxiety in some participants and subsequently affect outcome measurements. Randomisation of left and right lower limbs by limb dominance for exsanguination method was performed to reduce the effect of limb dominance on pain perception. Blinding of researchers was carried out to reduce observer bias.

With the duration of tourniquet inflation being 20 min, results from this study are limited in their external validity to relatively short lower limb operations. Another factor limiting clinical applicability of our results is the use of local and regional blocks in practice, such as spinal or epidural block in the setting of knee arthroplasty, which decrease pain perception from the tourniquet for a short while in the postoperative period. Another limitation of this study includes the interactions between the two lower limbs, with one potentially acting as a distractor to the other and augmenting pain perceptions. Studies have shown that a noxious stimulus can increase the pain threshold for a second, separate noxious stimulus [29, 30]. One’s selective attention ability and negative pain-related cognition like pain catastrophising are two further factors affecting perceived pain levels [30] given the simultaneous stimuli in this study.

## Conclusion

This randomised controlled trial investigated of impact of exsanguination technique in the use of surgical tourniquets of the lower limb. We found that there was a significant difference in pain levels between Esmarch exsanguination or exsanguination by elevation alone. These results suggest that pain implications may be a factor to consider in the choice of exsanguination technique prior to tourniquet application, in addition to surgeon preference and patient-specific factors, as the Esmarch bandage demonstrated decreased pain levels compared to elevation. This study contributes insights into the management of tourniquet-associated pain, a critical aspect of patient care in orthopaedic surgery. Future research could explore additional variables that might influence pain or examine surgical outcomes to further enhance patient care in surgical settings.
